# Endovascular interventional therapy and classification of vertebral artery dissecting aneurysms

**DOI:** 10.3892/etm.2014.1961

**Published:** 2014-09-12

**Authors:** YIHUA WANG, CUIPING ZHAO, XIAOGUANG HAO, CHENGWEI WANG, ZHIGANG WANG

**Affiliations:** 1 Department of Neurosurgery, Qilu Hospital of Shandong University, Qingdao, Shandong 266000, P.R. China; 2 Department of Neurosurgery, The Second Hospital of Shandong University, Jinan, Shandong 250033, P.R. China; 3 Department of Neurology, Qilu Hospital of Shandong University, Qingdao, Shandong 266000, P.R. China

**Keywords:** endovascular interventional therapy, vertebral artery, dissecting aneurysms

## Abstract

The current study aimed to summarise the clinical features and classifications of vertebral artery dissecting aneurysms (VADAs) to optimise strategies for endovascular interventional therapy. The clinical features and results of 31 inpatients with VADA were retrospectively analysed. The aneurysms were classified according to their location and association between the aneurysm and posterior inferior cerebellar artery (PICA), and into subtypes according to the developmental state of the contralateral vertebral artery. Different endovascular interventional therapy strategies were selected for each classification. Three types of aneurysm with two subtypes each were identified. An aneurysm located distally to the PICA was termed type I (10/31 patients). Aneurysms with a contralateral vertebral artery were denoted as subtype a (type Ia, 6/31 patients) and aneurysms with hypoplasia of the contralateral vertebral artery were denoted as subtype b (type Ib, 4/31 patients). An aneurysm located at the origin of the PICA was termed type II (13/31 patients), with seven cases classified as IIa and six cases as IIb. An aneurysm located proximally to the PICA was termed type III (8/31 patients), with five cases classified as IIIa and three cases as IIIb. Among the 31 patients, 18 received stent-assisted coiling, two received coiling, 10 received coiling with parent artery occlusion and one patient received conservative treatment. Among the 31 patients with VADA, 21 were occluded completely, nine were partially occluded and one was not occluded. One patient developed a coma following coiling; however, the other 30 patients recovered well. Thus, the classification of an aneurysm based on its location and the developmental state of the contralateral vertebral arteries appears to be an effective and safe approach for the selection of appropriate endovascular interventional therapy strategies.

## Introduction

Vertebral artery dissecting aneurysm (VADA) represents the underlying aetiology in a significant number of posterior circulation ischaemic strokes and subarachnoid haemorrhages (SAHs). When the lesions rupture and cause SAH, the percentage of rebleeding of these lesions is up to 30% (1,2). Thus, early treatment is strongly recommended. The standard treatment option is the complete occlusion of the dissected lesion through surgical or endovascular procedures. As the surgical approach is associated with a high incidence rate of treatment-associated mortality and morbidity, endovascular procedures are favoured in the treatment of VADA (3–5).

Endovascular treatment of VADA is complex and proximal occlusion of the parent artery and embolisation of the dissected segment have been proven to be optimal methods for VADA treatment (6). However, a number of more complex cases of VADA cannot be treated with proximal occlusion due to parent vessel sacrifice. Thus, parent vessel occlusion or stent-assisted coil embolisation has been utilised to treat these lesions. Treatment with a combination of stents and coils or with a stent or coil alone has been described in a number of patients (7–9). This treatment requires different therapeutic strategies due to the various states of the contralateral vertebral arteries or the location of the aneurysm in relation to the posterior inferior cerebellar artery (PICA). However, to the best of our knowledge, the classification of VADA on the basis of the state and location of bilateral vertebral arteries is unavailable. Thus, the current study aimed to classify VADAs by analysing the clinical features of the aneurysm, particularly its location and the state of the contralateral vertebral artery, in order to guide the optimal treatment strategy for these lesions.

## Materials and methods

### Patients

Between January 2004 and December 2011, 31 patients with VADA underwent endovascular treatment at the Department of Neurosurgery of the Second Hospital of Shandong University (Jinan, China). A total of 20 males and 11 females were included in the study group, with ages ranging from 25 to 73 years (mean age, 42.3 years). A total of 25 patients had hypertension and four had head or neck trauma. Approval was obtained from the Institutional Review Boards of the Second Hospital of Shandong University and Qilu Hospital of Shandong University (Qingdao, China) to study the use of coiling or stent-assisted coiling to treat VADA. The current study was conducted in accordance with the Declaration of Helsinki and with approval from the Ethics Committee of Shandong University. Written informed consent was obtained from all participants.

### Clinical features

A total of 23 patients with ruptured aneurysm presented with sudden headaches and computed tomography (CT) scans (SOMATOM Sensation 64; Siemens Medical Systems, Erlangen, Germany) revealed SAH. Among these patients, eight presented a coma and nine had dysphagia or another cranial nerve manifestation. Eight patients had headaches, vertigo and an unsteady gait, but no haemorrhage was detected by the CT scan. Only one patient presented brain stem infarction upon magnetic resonance imaging (MRI) examination (Magnetom Avanto; Siemens Medical Systems).

### Angiographic results and classification

All patients agreed to undergo CT scans and digital subtraction angiography (DSA; Innova 3100; GE Healthcare, Waukesha, WI, USA) for diagnosis. Angiograms were assessed for size, shape and location of VADA with respect to the PICA. The aneurysms were classified into three types on the basis of the location of the aneurysm in relation to the PICA: type I aneurysms, located distally to the PICA; type II aneurysms, located at the PICA origin and; type III aneurysms, located proximally to the PICA. Each type of aneurysm was further divided into two subtypes according to the developmental state of the contralateral vertebral artery. Subtype a included well-developed contralateral vertebral arteries and a guaranteed posterior circulation blood supply following the occlusion of the ipsilateral vertebral artery. Subtype b included contralateral vertebral arteries that were hypoplastic and would provide an inadequate posterior circulation blood supply following ipsilateral vertebral artery occlusion.

### Endovascular interventional therapy

Patients were placed under a state of general anaesthesia. Following a standard Seldinger puncture, a 6F introducer sheath (Cordis, Bridgewater, NJ, USA) was placed in the right femoral artery with heparinisation (by administrating heparin 2–3 mg/kg). Following the placement of a 6F guiding catheter (Cordis) in the ipsilateral vertebral artery at the level of the second cervical vertebrae, a roadmap was produced and an SL-10 microcatheter (Boston Scientific Corp, Natick, MA, USA) was delivered into the aneurysm. The aneurysm was embolised by appropriate micro-coils according to the shape and size of the aneurysm.

A 6F introducer sheath was placed in the right femoral artery and a 5F introducer sheath was placed in the left femoral artery. Following placement of the 6F guiding catheter into the ipsilateral vertebral artery, occlusion tests were carried out with an occlusion balloon to analyse whether the blood supply was adequate. The aneurysm was partially embolised with coiling after the microcatheter was navigated. The ipsilateral parent vertebral artery was subsequently occluded with coiling under the condition that PICA could obtain a sufficient blood supply from the ipsilateral or contralateral vertebral arteries.

Patients scheduled for stent-assisted coiling received oral aspirin (300 mg daily) and oral clopidogrel (75 mg daily) for three days to one week prior to the procedure. Patients undergoing emergency procedures received combined antiplatelet therapy (oral clopidogrel 300 mg and aspirin 300 mg) on the day of surgery.

A 4.5×22 or 4.5×28 mm Enterprise stent (Codman & Shurtleff, Raynham, Massachusetts, USA) was used to cover the neck of the aneurysm following placement of a Prowler-plus microcatheter (Codman) in the dissecting aneurysm. Coiling was subsequently performed and the stent was finally released.

### Post-operative treatment

Patients with SAH or intraventricular haemorrhage were given a lumbar puncture or catheter following surgery. External ventricular drainage was performed on patients in a coma with intraventricular haemorrhage. Patients with stent-assisted coiling were administered antiplatelet agents (clopidogrel 75 mg and aspirin 300 mg daily) for three months. After three months, the antiplatelet agents were adjusted according to the patients’ condition. All patients were reviewed for post-operative angiography at one, three and six months, and one year.

## Results

### Primary classification

A total of 10 patients had a type I aneurysm (10/31 patients), of which six patients were type Ia and four patients were type Ib. A total of 13 patients were type II (13/31 patients), of which seven were type IIa and six patients were type IIb. A total of eight patients were type III (8/31 patients), of which five patients were type IIIa and three patients were type IIIb (Table I).

### Endovascular interventional therapy

Among the 31 patients, 18 patients underwent stent-assisted coiling, two cases received coiling only, 10 received coiling with parent artery occlusion and one case was treated conservatively. Among the 31 patients with VADA, 21 were embolised completely, nine were partially embolised and one was not embolised. One patient developed a coma following coiling and the rest of the patients recovered well without any neurological deficits. No mortalities were reported among the 31 patients.

### Treatment approach according to classification

The approaches used in the endovascular interventional treatment were selected carefully according to the classification of the VADA (Table II).

**Figure 1:**
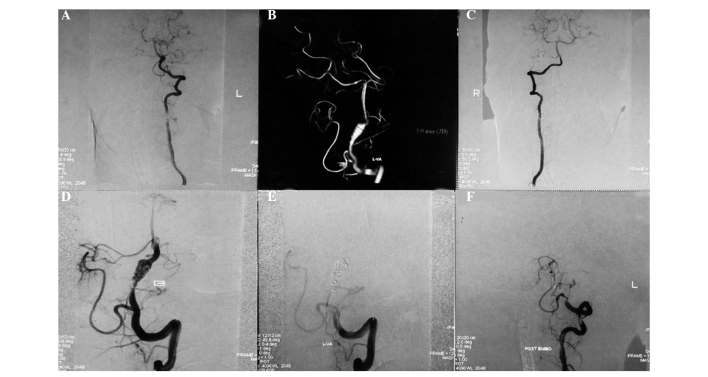
(A) Digital subtraction angiography (DSA) and (B) 3D-DSA images showing a well-developed type Ia dissecting aneurysm of the left vertebral artery located distally to the posterior inferior cerebellar artery (PICA) and the contralateral vertebral artery. (C) DSA images of the right vertebral artery and (D) an amplified image of the left vertebral artery. The aneurysm was treated with coiling combined with left vertebral artery occlusion. The post-treatment angiograms show complete obliteration of the aneurysm, (E) occlusion of the distal part of the left vertebral artery and (F) a well-filled PICA.

**Figure 2:**
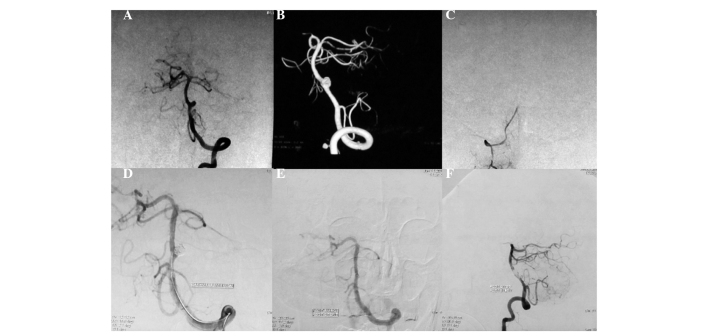
(A) Digital subtraction angiography (DSA) and (B) 3D-DSA images showing a type Ib dissecting aneurysm of the left vertebral artery located distally to the posterior inferior cerebellar artery and the contralateral vertebral artery, exhibiting hypoplasia. DSA images showing the (C) right vertebral artery and (D) stent-assisted coiling treatment of the left vertebral dissecting aneurysm. The post-treatment angiograms show (E) complete obliteration of the aneurysm and (F) that the left vertebral artery was filled well.

**Figure 3:**
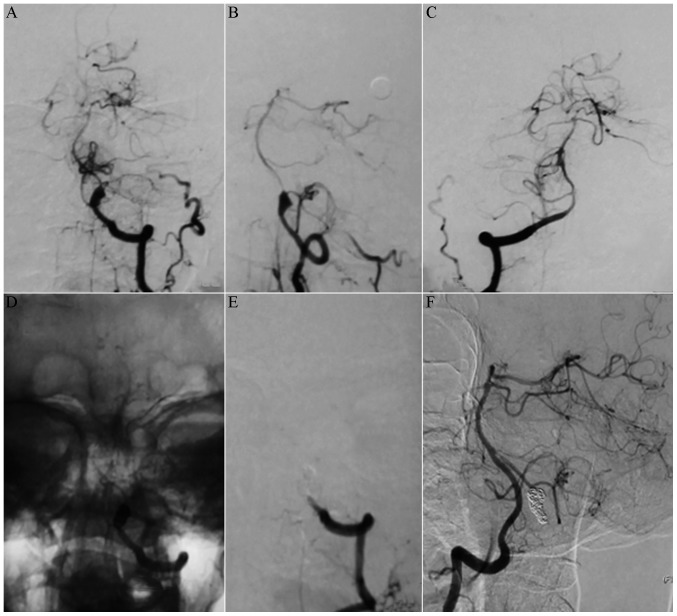
(A) Digital subtraction angiography (DSA) and (B) oblique DSA images showing a well-developed type IIa dissecting aneurysm of the left vertebral artery located at the origin of the posterior inferior cerebellar artery (PICA) and the contralateral vertebral artery. (C) DSA image of the right vertebral artery and (D) negative image showing the left vertebral dissecting aneurysm following coiling. The post-treatment angiograms show complete obliteration of the aneurysm, (E) occlusion of the left vertebral artery and (F) the PICA filled with blood from the right vertebral artery.

**Figure 4:**
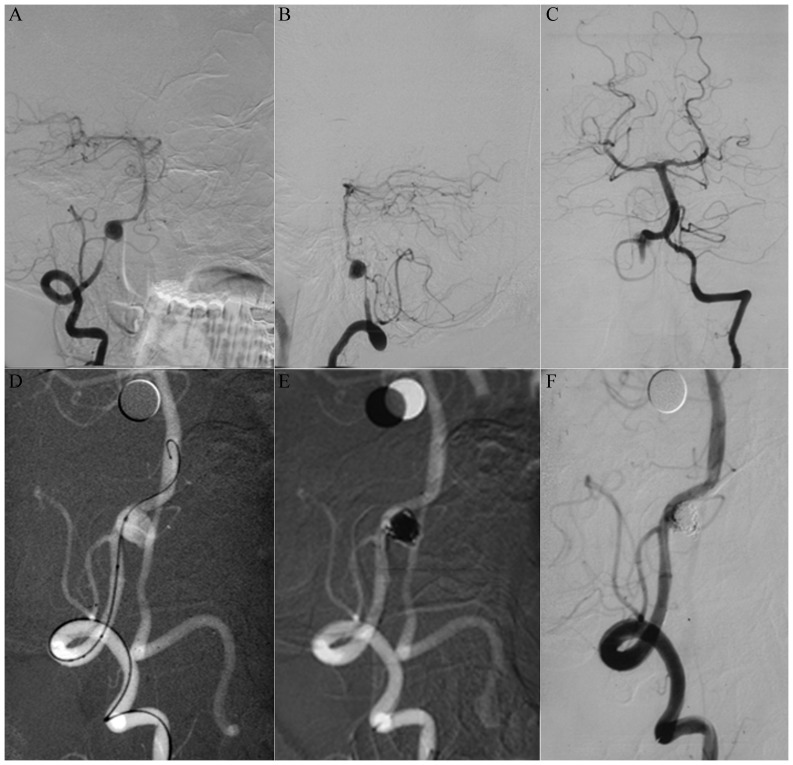
(A) Digital subtraction angiography (DSA) and (B) oblique DSA images showing a well-developed type IIa dissecting aneurysm of the right vertebral artery located at the origin of the posterior inferior cerebellar artery and the contralateral vertebral artery. DSA images showing the (C) left vertebral artery and (D) stent-assisted coiling treatment of the right vertebral artery. The post-treatment angiograms show (E) complete obliteration of the aneurysm and (F) that the right vertebral artery was filled well.

**Figure 5:**
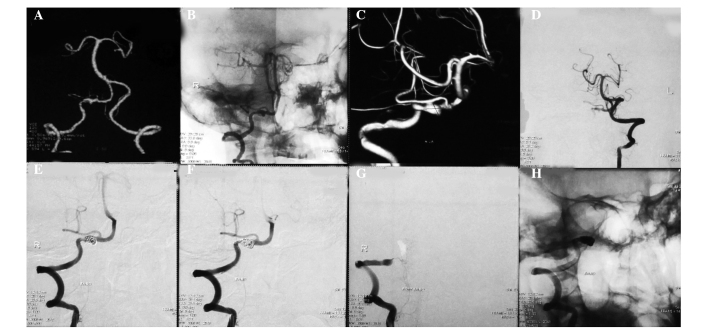
(A) Computed tomography angiography image showing the bilateral vertebral artery. (B) Negative and (C) 3D digital subtraction angiography (DSA) images showing a well-developed type IIIa dissecting aneurysm of the right vertebral artery located proximally to the posterior inferior cerebellar artery and the contralateral vertebral artery. The aneurysm was treated with coiling combined with right vertebral artery occlusion. (D) DSA image of the left vertebral artery. The post-treatment angiograms show (E) complete obliteration of the aneurysm and (F and G) occlusion of the distal part of the right vertebral artery. (H) Negative image of the right vertebral artery following treatment.

**Figure 6:**
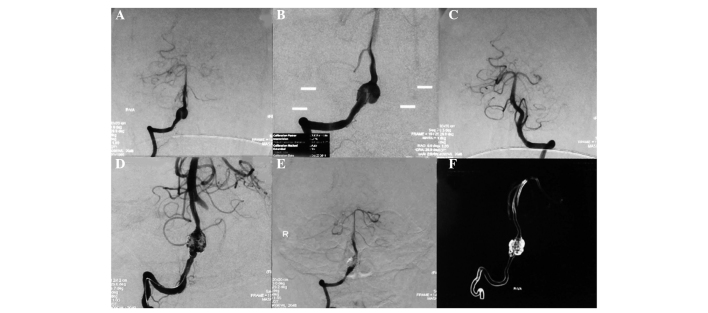
(A) Digital subtraction angiography (DSA) and (B) amplified DSA images showing a well-developed type IIIa dissecting aneurysm of the right vertebral artery located proximally to the posterior inferior cerebellar artery and the contralateral vertebral artery. DSA images showing the (C) left vertebral artery and (D) stent-assisted coiling treatment of the right vertebral dissecting aneurysm. The post-treatment angiograms show (E) complete obliteration of the aneurysm and (F) that the right vertebral artery was filled well.

Among the 10 patients with type I aneurysms, four patients with type Ia received coiling with parent artery occlusion (Fig. 1), one patient with type Ia received stent-assisted coiling, three patients with type Ib received stent-assisted coiling (Fig. 2), one patient with type Ib received coiling only and one patient with type Ib was treated conservatively. Among the 13 patients with type II aneurysms, two patients with type IIa received coiling with parent artery occlusion (Fig. 3), five patients with type IIa received stent-assisted coiling (Fig. 4) and all six patients with type IIb received stent-assisted coiling. Among the eight patients with type III aneurysms, four patients with type IIIa received coiling with parent artery occlusion (Fig. 5), one patient with type IIIa received stent-assisted coiling (Fig. 6), two patients with type IIIb received stent-assisted coiling and one patient with type IIIb received coiling only.

Of the 17 patients classified as subtype a, 10 patients received coiling with parent artery occlusion and seven patients received stent-assisted coiling. Of the 14 patients classified as subtype b, 11 patients received stent-assisted coiling, two patients received coiling only and one patient was treated conservatively.

## Discussion

The incidence rate of VADA is relatively low and the annual incidence rate has been reported to be 1/100,000 cases in the USA and 1.5/100,000 cases in France (10–12). VADA is common in adults, particularly those aged 40–50 years. The onset manifestation is SAH or cerebral ischaemia. SAH usually occurs in patients with intracranial aneurysm whereas ischaemic attack occurs in patients with extracranial aneurysms (12,13). A local mass effect with a giant aneurysm has also been detected. SAH caused by VADA explains ~10% of all causes of non-traumatic SAH with a reported mortality rate ranging from 19 to 83% (14).

VADA is frequently ruptured due to severe SAH with catastrophic neurological outcomes and a high incidence rate of recurrent haemorrhage (1,2,15). Mizutani *et al* retrospectively analysed 42 patients with recurrent haemorrhage caused by the rupture of VADA and revealed that 40.5% of recurrent haemorrhages occurred within 24 h and that 57.1% occurred one week following the first haemorrhage (16). Yamada *et al* studied the conservative management of 24 patients with VADA and determined that 58% of these cases occurred with recurrent haemorrhage and 46% succumbed due to catastrophic rebleeding (17).

DSA remains the gold standard in diagnostic imaging. The classical manifestations of VADA in DSA are as follows: pearl and string, double lumen, rosette or a simple fusiform dilation, as well as a delayed clearance of dilatation or a false lumen (18–20). The treatment of VADA is a technical challenge due to the histopathological features and special localisations of aneurysms. Endovascular intervention therapy, rather than conventional surgery, is preferred in the treatment of certain cases of VADA as surgical trauma is avoided in the former (2,13). The dissecting aneurysm does not have a real neck and conventional clipping does not manage the aneurysm successfully. Endovascular intervention therapy is becoming the primary option for VADA treatment (21). The vertebral artery should be occluded or preserved when the aneurysm is treated with the intervention technique. Occlusion of the vertebral artery may bring about a low rate of recurrence, or prevent recurrence. However, this occlusion affects the ipsilateral vertebral blood supply, which may result in severe complications, including ischaemic lesioning of the brainstem. Certain factors affecting contralateral vertebral arteries, including the condition of the artery and the age of the patient, should be carefully considered.

In the current study, 17 patients exhibited a subtype a aneurysm. Of these, 10 patients received coiling with parent artery occlusion (58.8%) and seven patients received stent-assisted coiling (41.2%). All patients who were treated with coiling combined with parent artery occlusion were not recurrent and had no ischaemia in the posterior blood circulation in the follow-up review. In the seven patients with stent-assisted coiling, the vertebral artery was preserved due to the patients’ refusal to undergo vertebral occlusion in two cases and due to intolerance to the occlusion test in two cases; the three remaining three patients were younger patients in which preservation of the artery was selected to avoid potential ischaemic events. However, in a later review, one of the seven patients developed a mild recurrence at the aneurysm neck and was subjected to repeated embolism. A total of 14 patients exhibited subtype b aneurysm and the vertebral artery was not occluded due to hypoplasty of the contralateral vertebral artery. Eleven of the 14 patients accepted stent-assisted coiling, of which eight were occluded completely and three were partially embolised. In patients with complete occlusion, one experienced recurrence during the follow-up period and one patient developed a mild recurrence at the aneurysm neck. In patients with partly occluded aneurysms, one patient was observed to have an enlarged aneurysm in the follow-up angiography. Two patients of subtype b were treated with coiling directly since the neck of aneurysm was relatively narrow and one patient selected conservative treatment for financial reasons. Stent-assisted coiling was the preferred treatment for type II patients to avoid affecting the PICA since the aneurysms were located at the origin of the PICA.

Positive therapeutic effects for VADA have been attained with the development of the intervention technique. However, further studies are required to optimise the therapy strategy in order to improve the selection of the most appropriate therapies for individual patients (22–24). The classification of VADA may enable the therapy to be optimized. Coiling with parent artery embolisation is an effective choice for patients classified as subtype a since this method is able to avoid recurrence and potential ischaemic events. The vertebral artery should be preserved in young patients and patients who cannot fully tolerate an occlusion experiment prior to embolisation. For patients classified as subtype b, preservation of the vertebral artery is required and stent-assisted coiling is preferred despite ready recurrence. Patients should be regularly reviewed and embolism performed if required. For patients with a type II aneurysm, the parent artery should be preserved to prevent affects on the PICA. In the future, flow-diverting devices (dense stent-mesh) are likely to be an effective choice for large dissection aneurysms.

Thus, the classification of an aneurysm based on its location and the developmental state of the contralateral vertebral arteries appears to be an effective and safe approach for the selection of appropriate endovascular interventional therapy strategies.

## Figures and Tables

**Table I:** Primary classification of vertebral artery dissection aneurysm.

Type	Subtype	Cases (n)	Proportion (%)
I	Ia	5	16.1
	Ib	5	16.1
II	IIa	7	22.6
	IIb	6	19.4
III	IIIa	5	16.1
	IIIb	3	9.7

**Table II:** Classification and treatment strategy.

Type	Coiling with parent artery embolization (%)	Stent-assisted coiling (%)	Coiling (%)	Conservative treatment (%)
I type	4 (40)	4 (40)	1 (10)	1 (10)
Ia	4 (80)	1 (20)		
Ib		3 (60)	1 (20)	1 (20)
II type	2 (15.4)	11 (84.6)		
IIa	2 (28.6)	5 (71.4)		
IIb		6 (100)		
III type	4 (50)	3 (37.5)	1 (12.5)	
IIIa	4 (80)	1 (20)		
IIIb		2 (66.7)	1 (33.3)	
Subtype a	10 (58.8)	7 (41.2)		
Subtype b		11 (78.6)	2 (14.3)	1 (7.1)
